# Nutrition facts labels: who is actually reading them and does it help in meeting intake recommendations for nutrients of public health concern?

**DOI:** 10.1186/s12889-023-16859-2

**Published:** 2023-10-07

**Authors:** Maximilian Andreas Storz

**Affiliations:** Department of Internal Medicine II, Centre for Complementary Medicine, Faculty of Medicine, University of Freiburg, Freiburg University Hospital, Freiburg, Germany

**Keywords:** Dietary fiber, Dietary intake recommendations, Public health nutrition, Nutrition facts panels, Dietary choices, Dietary Guidelines for Americans, Calcium, Potassium

## Abstract

**Background:**

Fiber, potassium and calcium are nutrients of public health concern and their intakes in the United States are alarmingly low. The usage of nutrition facts labels has been reported to increase the odds for dietary reference intake of fiber in some studies. The overall evidence, however, is mixed, as some studies suggested that nutrition facts panels have little to no effect on average measures of diet quality. Here, we investigated whether the usage of nutrition facts labels was associated with meeting U.S. intake recommendations for three nutrients of public health concern: fiber, potassium and calcium.

**Methods:**

We used cross-sectional multistage, stratified, clustered and probability sampling design data from the U.S. National Health and Nutrition Examination Surveys 2017–2020 cycle. The sample included 5,416 individuals aged 20 years or older, which may be extrapolated to represent 146,841,866 US Americans. Nutrient intakes were compared among individuals reading nutrition facts panels “frequently”, “sometimes” or “rarely” using applied survey data analyses techniques (including multivariate logistic regression and marginsplots).

**Results:**

We observed substantial sociodemographic differences between the three groups. Frequent readers were significantly more likely to be female and had higher educational levels. On average, they were also significantly older as compared to rare readers. Fiber intake in g/d was highest in frequent readers (17.09) and lowest in rare readers (14.64). The proportion of participants that met dietary fiber intake recommendations was almost four times higher in the frequent readers group (12.69%) as compared to the rare readers group (3.69%). In a bivariate logistic regression model, frequent label reading significantly increased the odds for meeting the fiber recommendations in Dietary Guidelines for Americans (OR: 2.15, *p* < 0.001). Rarely reading labels decreased the odds (OR: 0.57, *p* = 0.003). These odds remained essentially unchanged after adjusting for sociodemographic covariates, diabetes status and body mass index (OR: 1.84, *p* = 0.004; and OR: 0.62, *p* = 0.022).

**Conclusions:**

Nutrition facts panel reading associates with fiber intake. Our findings have potential implications for public health nutrition strategies that may center around educational work.

**Supplementary Information:**

The online version contains supplementary material available at 10.1186/s12889-023-16859-2.

## Background

An optimal fiber intake is of paramount importance for human health as it may help to improve glycemic control and to reduce low low-density lipoprotein cholesterol levels [1, 2]. Fibers may also improve laxation, thereby preventing constipation and other large bowel diseases [3–5].

Despite these benefits, fiber intakes in the United States are alarmingly low, with long-term implications for public health [6, 7]. On average, American adults eat only between 10 and 15 g of total fiber per day. The most recent Dietary Guidelines for Americans (DGA), however, recommend an intake of at least 14 g per 1000 kcal per day [8]. Depending on energy intake, this translates into a minimum recommended intake of approximately 28 g of fiber in women and 35 g in men per day.

The “American fiber gap” constitutes a serious public health problem that may be aggravated with the re-emergence of low-carbohydrate diets, which may even further decrease fiber consumption [9, 10]. Like potassium and calcium, fiber is an underconsumed nutrient of public health concern in the United States and new strategies to increase the intakes of these important nutrients are urgently warranted [11]. The average daily potassium intake of the U.S. population aged 2 years and older was 2496 mg based on data from 2017–2018, and thus way below the recommended intake [12]. As for calcium, data also points at an insufficient intake in several population groups, particularly in older adults [13].

**Fig. 1:**
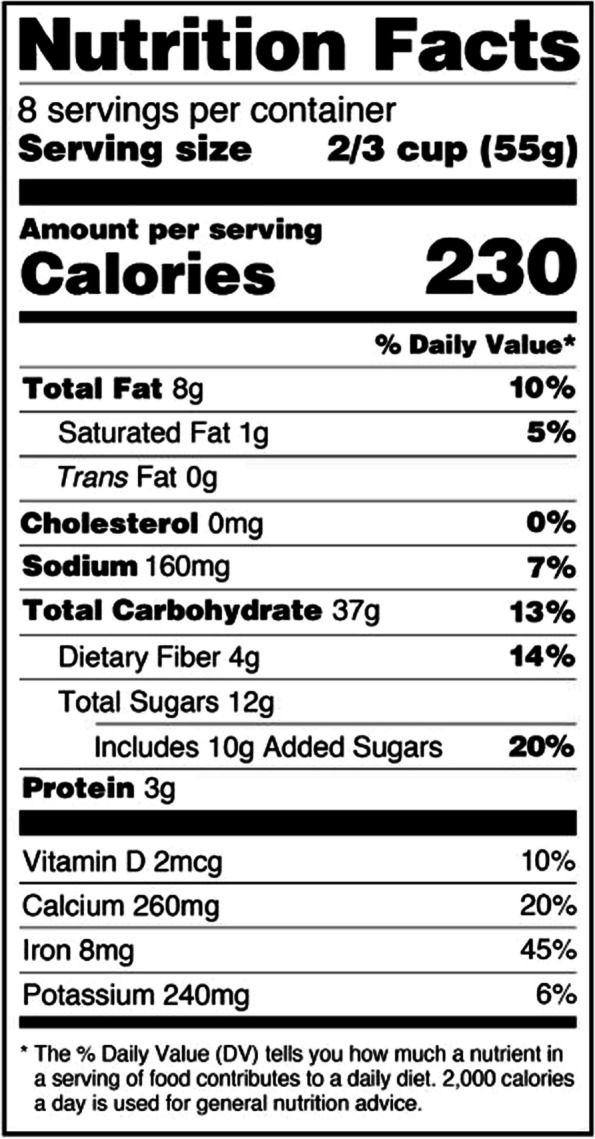
Nutrition facts label example from the U.S., modified from the FDA homepage (https://www.fda.gov/food/food-labeling-nutrition/changes-nutrition-facts-label), published under a Public Domain (19 September 2023)

Nutrition facts labels (as shown in Fig. 1) could potentially help to increase the intakes of these nutrients of public health concern in the in the U.S. population. Redesigned by the Food and Drug Administration (FDA) a few years ago, nutrition facts labels include information on dietary fiber, potassium and calcium content on a mandatory basis [14]. The current FDA reference value for dietary fiber is 28 g per day.

A major goal of nutrition facts labelling is to make informed food choices contributing to lifelong healthy eating habits. By breaking down the amount of calories, carbohydrates, fat, fiber, protein, and vitamins per serving of a specific food, comparison of the nutrient content of similar products should be facilitated [15].

Yet questions arise as to who is actually reading nutrition facts labels [16], and whether they are helpful in increasing the intakes of specific nutrients. In this context, Kim et al. reported a positive association between nutrition label reading and an increased intake of fiber in Korean men but not in women [17]. Nutrition label reading significantly increased the odds for dietary reference intake of fiber (OR: 2.00, CI 1.23 to 3.26) in a large Korean sample. Whether these associations are transferable to U.S. adults on a national level, however, remains subject to a controversial debate [18, 19]. The evidence associating the usage of nutrition facts panels with a healthy diet is mixed and specific outcomes and impacts in the U.S. are less clear [16]. Significant gaps remain how nutrition label users compare on broad dietary outcomes, specific micronutrients and compliance with the DGA [16]. While some studies suggested that nutrition facts panels on packaged food products have little to no effect on average measures of diet quality [20, 21], other authors reported opposite findings [22–24].

To delve deeper into this topic, we used nationally representative data from some of the most recently available U.S.-based National Health and Nutrition Examination Surveys (NHANES) cycles and sought to answer the following questions: (a) Who is actually reading nutrition facts labels in the U.S. population, and (b): is reading of nutrition facts labels associated with meeting DGA recommendations of underconsumed nutrients of public health concern (fiber, potassium, calcium)?

## Methods

### Study population and design

Data for this analysis was obtained from the NHANES—an ongoing program of studies by the Centers for Disease Control and Prevention which has been designed to assess the health and nutritional status of the non-institutionalized population in the United States of America [25, 26]. Due to its complex multistage, stratified, clustered and probability sampling design, NHANES allows investigators to compute nationally representative nutritional status assessments. Characteristics and study design, including recruitment methods and program execution details, have been previously discussed [25, 26]. This analysis is based on data from the 2017 – 2020 NHANES cycle, which included 15,560 participants. NHANES has been approved by the National Center for Health Statistics (NCHS) and all participants gave written and oral consent to participate in the study [27].

### Usage of nutrition facts panel on food labels

Participants’ nutrition facts panel reading behavior was obtained from the NHANES consumer behavior phone follow-up module [28]. This NHANES section provides personal interview data on various dietary related consumer behavior topics. Data was collected at person-level, meaning that each participant answered the questions for herself/himself. Participants were shown an example of a food label that included a nutrition facts panel marked in yellow. Subsequently, participants were explained that a food label “is usually on the back or the side of a food package” and “consists of two parts”: the nutrition facts panel (listing the amount of calories, fat, fiber, carbohydrates and some other nutritional information) and a list of ingredients. Participants were then asked “How often do you use the Nutrition Facts panel when deciding to buy a food product?" with the following possible answer options: “Always”, “Most of the time”, “Sometimes”, “Rarely”, “Never” and “Never seen”. Participants who replied “always” or “most of the time” were combined into the “frequent readers” group, whereas those replying “rarely or never” where combined in the “rare readers” group. Participants that had never seen the label (*n* = 2) and participants with missing data (*n* = 1752) were excluded from the analysis.

### Underconsumed nutrients of public health concern

The following underconsumed nutrients of public health concern were examined: fiber, potassium and calcium [8]. Total fiber intake in g/d was obtained from the NHANES dietary data module and computed in g/1000 kcal/d [29]. Potassium and calcium were obtained from the same module and displayed in mg/d or mg/1000 kcal/d. Two groups were constructed: participants that had a fiber intake of 14 g/d/1000 kcal or more (and who were thus in line with the aforementioned recommendations) and participants that did not meet this cut-off. In a similar style, two groups were constructed for potassium and calcium intake whereby age and sex-specific intake recommendations from the DGA were considered [8].

Only nutrients from foods and not from supplements was considered. Total energy in kcal/d was also obtained from this module. The nutrient intake assessment was conducted in person by trained dietary interviewers fluent in Spanish and English. It was based on a computerized 24 h dietary recall method to estimate energy and nutrient intake for all participants. The exact methods have been described elsewhere in detail [30, 31].

### Covariates

To delve deeper into the question “who is actually reading nutrition facts panels?”, we included the following sociodemographic covariates: age in years (continuous variable), sex (categorical variable: female, male); race/ethnicity (categorical variable: Mexican American; Other Hispanic; Non-Hispanic White; Non-Hispanic Black; Other Race); marital status (married/living with partner; widowed/divorced/separated and never married), educational level (categorical variable: less than 9th grade; 9-11th grade; high school graduate/GED; some college or AA degree; college graduate or above), and ratio of family income to poverty (categorical variable: < 1; ≥ 1 and < 2; ≥ 2 and < 3; ≥ 3). Additional covariates included body mass index (BMI) and diabetes status (as assessed by the question “Have you ever been told by a doctor or health professional that you have diabetes or sugar diabetes?”).

### Inclusion and exclusion criteria

Inclusion criteria were as follows: age ≥ 20 years, available demographic data, available nutrient intake data, plausible energy intake data (based on Willett’s criteria [32]), and no missing data to the respective questions on nutrition labels in the NHANES Consumer Behavior Phone Follow-up Module. All participants with missing data were excluded from the analysis.

### Statistical analysis

We used Stata version 14 (StataCorp, College Stadion, TX, USA) for statistical analyses and analysed the merged dataset based on Heeringa’s applied survey data analysis techniques [33]. We used appropriate sample weights provided by the NHANES to account for the complex, multistage, probability sampling design and paid special attention to the data analysis instructions for the 2017–2020 pre-pandemic cycle.

Continuous variables were described with their mean and corresponding standard error in parentheses when normally distributed. Categorical variables were described as weighted proportions with their corresponding standard error. As described previously, all weighted proportions were carefully checked for their reliability based on the recent NCHS data presentation standards [34, 35].

For categorical variables, we used Stata’s design-adjusted Rao–Scott test to explore potential associations between fiber intake and nutrition facts panel reading status as well as other sociodemographic variables. Multivariate linear regression analyses (followed by adjusted Wald tests) were used to test for potential differences in continuous variables across groups.

Finally, we ran multivariable logistic regression models to examine the associations of nutrition facts panel usage and the intake of nutrients of public health concern. The dependent binary variable indicated whether the DGA intake recommendation for a specific nutrient were met or not met. Three models were constructed for each nutrient under discussion. After assessing crude associations, we adjusted for a variety of sociodemographic covariates in model 2. In addition to that, we additionally adjusted for total energy intake, BMI and diabetes status in model 3. All models were constructed in accordance with West, Berglund, and Heeringa’s recommendations for applied survey data analyses [33]. Potential covariates were chosen based on initial exploratory bivariate analyses and based on previous publications in the field [36–38].

For significant outcomes, we also ran linear regression models and estimated mean adjusted nutrient intakes by label reading group. Post regression, we used marginsplots to graph statistics from fitted models. Statistical significance was determined at α = 0.05.

## Results

**Fig. 2:**
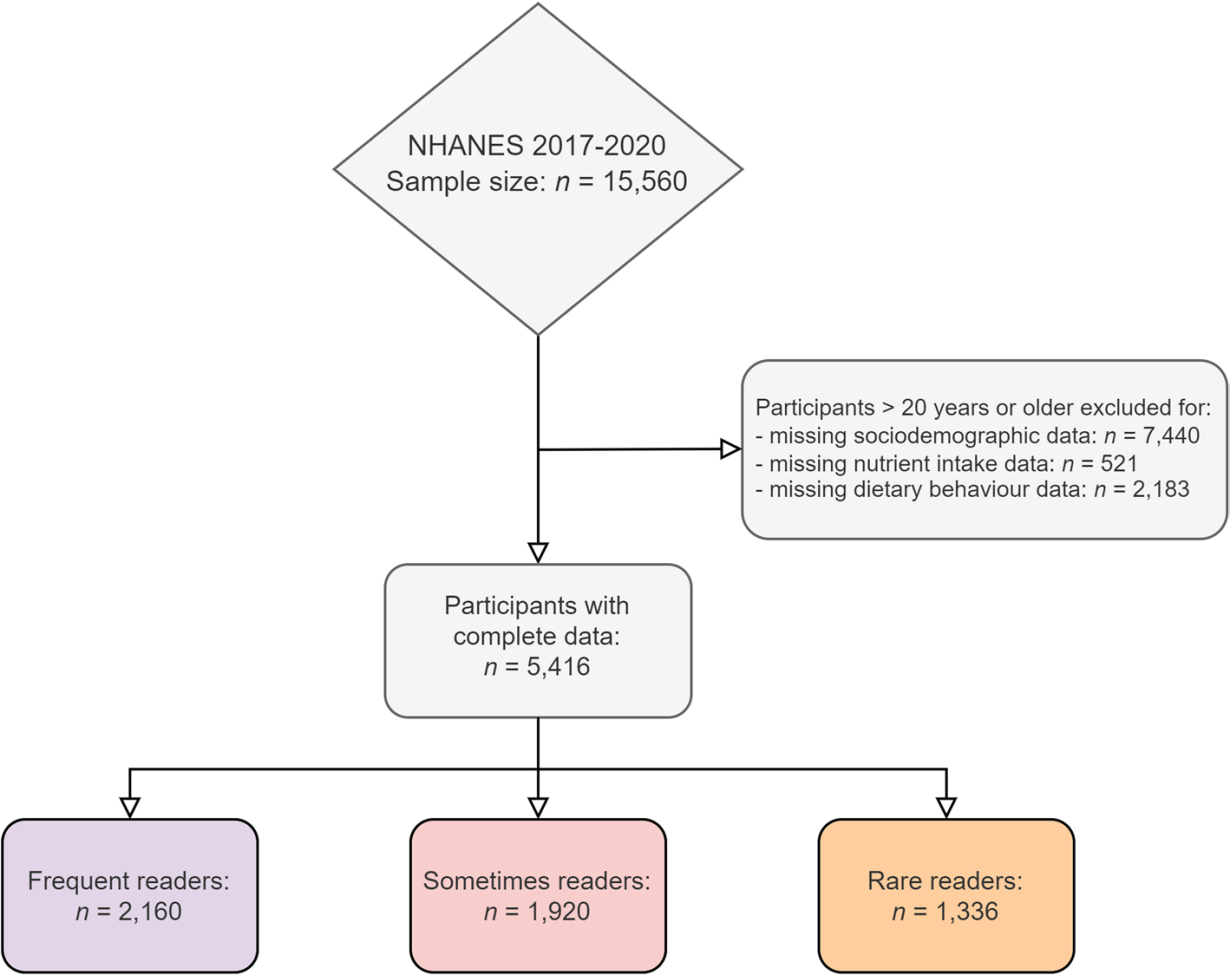
Participant inclusion flowchart

The total sample for this analysis included = 5,416 individuals (see Fig. 2). The frequent readers group comprised *n* = 2160 participants, the sometimes readers group included *n* = 1,920 participants. The rare readers group was the smallest group with *n* = 1,336 participants. The total sample may be extrapolated to represent 146,841,866 US Americans.

Table 1 shows sample characteristics by nutrition facts panel reading behavior. Significant intergroup differences were found with regard to sex and age. Frequent readers were significantly more likely to be female, whereas rare readers were significantly more likely to be male. Rare readers were significantly younger as compared to the other groups. Significant intergroup differences were also found with regard to marital status, race/ethnicity and education level. The weighted proportion of participants with a college degree or higher was significantly higher in frequent readers as opposed to the other two groups. We also observed significant intergroup differences with regard to BMI and diabetes status. BMI was highest in the sometimes readers group and lowest in rare readers. The proportion of individuals with diabetes was highest among the frequent reader group.

**Table 1:** Sample characteristics by label reading behaviour

	**Frequent Readers: *n* = 2,160**		**Sometimes Readers: *n* = 1,920**		**Rare Readers: *n* = 1,336**		***p*-value**
	**Weighted Proportion (%)**	**SE**	**Weighted Proportion (%)**	**SE**	**Weighted Proportion (%)**	**SE**	
**Sex**							*p* < 0.001^b^
Male	44.78	1.24	49.65	1.48	65.15^e^	2.04	
Female	55.22	1.24	50.35	1.48	34.85^e^	2.04	
**Age (years)**	51.82	0.80	49.50	0.76	46.22	0.58	*p* < 0.001^c^
**Marital status**							*p* = 0.004^b^
Married/Living with Partner	63.80	1.76	64.16	2.30	59.55	1.93	
Widowed/Divorced/Separated	21.35	1.31	16.84	1.02	18.40^e^	1.88	
Never married	14.85	1.25	18.98	1.72	22.05^e^	1.64	
**Race/Ethnicity**							*p* < 0.001^b^
Mexican American	5.32	1.01	7.15	1.13	10.71^e^	1.50	
Other Hispanic	7.87	0.93	6.05	0.77	7.53	1.02	
Non-Hispanic White	66.74	2.69	66.96	2.78	60.37^e^	3.05	
Non-Hispanic Black	10.70	1.47	12.29	2.03	13.92^e^	1.58	
Other Race ^a^	9.37	1.37	7.55	1.02	7.47	0.86	
**Education Level**							*p* < 0.001^b^
Less than 9th grade	1.72	0.32	2.32	0.32	4.80^e^	0.66	
9-11th grade	3.91	0.41	5.87	0.70	10.36^e^	0.76	
High school graduate/GED ^d^	20.30	1.53	26.83	1.80	36.78^e^	2.21	
Some college or AA degree	30.98	1.59	31.17	2.16	32.73	1.64	
College graduate or above	43.09	2.82	33.81	2.79	15.33^e^	1.76	
**Ratio of family income to poverty**							*p* < 0.001^b^
< 1	7.80	0.92	9.88	1.19	16.65^e^	1.17	
≥ 1 and < 2	14.00	1.07	14.53	1.31	21.48^e^	2.17	
≥ 2 and < 3	13.00	1.34	14.65	1.70	16.49	1.29	
≥ 3	65.31	2.06	60.95	1.87	45.38^e^	2.86	
**Body mass index (kg/m**^**2**^**)**	29.73	0.26	30.47	0.33	29.07	0.26	*p* = 0.005^c^
**Diabetes status**							*p* = 0.024^b^
Yes	13.90	1.12	12.93	1.03	9.09^e^	1.08	
No	86.10	1.12	87.07	1.03	90.91^e^	1.08	

Weighted proportions. Total number of unweighted observations: *n* = 5,416. Continuous variables shown as mean (standard error). Categorical variables shown as weighted proportion (standard error). All weighted proportions can be considered reliable, as peer recent NCHS Guidelines. Percentages may not total 100 due to rounding

^a^Includes Multi-Racial

^b^Based on Stata’s design-adjusted Rao–Scott test

^c^Based on regression analyses followed by adjusted Wald tests

^d^Or equivalent

^e^Shows significant differences in the weighted proportions

Total energy intake in kcal/d and fiber intake also varied significantly across groups (Table 2). Energy intake was lowest in those frequently reading nutrition facts panels, whereas it was highest in those participants rarely reading labels. Total unadjusted fiber intake was highest in frequent readers and lowest in rare readers – despite the higher total energy intake in this group. This phenomenon was also reflected in the energy-adjusted intake values. For potassium and calcium, significant differences were “only” observed for energy adjusted values. Table 2 also shows that the proportion of participants not meeting the DGA intake recommendation was high for all nutrients, whereby fiber stood out with almost 96.50% in the “rare reader” group.

**Table 2:** Nutrient and energy intake by label reading behaviour

	**Frequent Readers: *n* = 2,160**		**Sometimes Readers: *n* = 1,920**		**Rare Readers: *n* = 1,336**		***p*-value**
	**Mean**	**SE**	**Mean**	**SE**	**Mean**	**SE**	
**Energy intake (kcal/d)**	1993.03	25.15	2170.51	28.43	2397.19	41.24	*p* < 0.001 ^a^
**Fibre intake (g/d)**	17.09	0.42	15.82	0.36	14.64	0.35	*p* < 0.001 ^a^
**Fibre intake (g/1000 kcal)**	8.86	0.18	7.60	0.16	6.30	0.17	*p* < 0.001^a^
**Potassium intake (mg/d)**	2614.55	35.19	2526.25	45.35	2518.33	44.51	*p* = 0.097^a^
**Potassium intake (mg/1000 kcal)**	1383.26	17.18	1209.61	17.86	1102.64	17.22	*p* < 0.001^a^
**Calcium intake (mg/d)**	907.89	13.43	899.91	23.96	913.52	20.03	*p* = 0.928^a^
**Calcium intake (mg/1000 kcal)**	475.44	7.56	431.47	7.97	419.25	17.40	*p* < 0.001^a^
	**Weighted Proportion (%)**	**SE**	**Weighted Proportion (%)**	**SE**	**Weighted Proportion (%)**	**SE**	
**Met DGA Fiber recommendations**							*p* < 0.001^b^
No	87.31	1.53	93.68	0.82	96.31^c^	0.64	
Yes	12.69	1.53	6.32	0.82	3.69^c^	0.64	
**Met DGA Potassium recommendations**							*p* = 0.085^b^
No	66.48	1.37	70.77	1.65	73.40^c^	1.50	
Yes	33.52	1.37	29.23	1.65	26.60^c^	1.50	
**Met DGA Calcium recommendations**							*p* = 0.048^b^
No	73.30	1.14	73.16	1.84	67.45^c^	1.76	
Yes	26.70	1.14	26.84	1.84	32.55^c^	1.76	

Total number of unweighted observations: *n* = 5,416. Continuous variables shown as mean (standard error). Categorical variables shown as weighted proportion (standard error). All weighted proportions can be considered reliable, as peer recent NCHS Guidelines. Percentages may not total 100 due to rounding

^a^Based on regression analyses followed by adjusted Wald tests

^b^Based on Stata’s design-adjusted Rao–Scott test

^c^Shows significant differences in the weighted proportions

Table 3 shows the results of the multivariate logistic regression models for fiber. In a crude model (model 1), frequent label reading significantly increased the odds for meeting the DGA fiber recommendations (OR: 2.15, *p* < 0.001), whereas rarely reading labels decreased the odds (OR: 0.57, *p* = 0.003). In model 2, these odds remained essentially unchanged after adjusting for sociodemographic covariates. In model 3, in which we additionally adjusted for total energy intake, BMI and diabetes status, the odds for meeting the DGA fiber recommendations slightly decreased in the frequent reader group (OR: 1.82, *p* = 0.004). At the same time, the odds in rare readers slightly increased (OR: 0.62, *p* = 0.022). A fourth model additionally adjusting for income resulted in no significant model improvements (not shown).

**Table 3:** Multivariate logistic regression models examining potential associations between nutrition panel reading frequency and fibre intake

**Independent variables**	OR	**CI**	**p**	OR	**CI**	**p**	OR	**CI**	**p**
	Model I			Model 2			Model 3		
**Panel reading frequency**									
Frequent readers	2.15	[1.47; 3.16]	< 0.001	1.97	[1.31; 2.96]	0.002	1.82	[1.23; 2.69]	0.004
Sometime readers	-	-	-	-	-		-	-	
Rare/never readers	0.57	[0.40; 0.81]	0.003	0.65	[0.45; 0.93]	0.020	0.62	[0.41; 0.93]	0.022
**Sex**									
Female				1.48	[1.02; 2.17]	0.042	0.97	[0.69; 1.38]	0.875
Male				-	-	-	-	-	-
**Age**									
In years				1.01	[0.99; 1.02]	0.058	1.01	[1.00; 1.02]	0.048
**Ethnicity**									
Mexican American				2.86	[1.56; 5.25]	0.001	3.36	[1.72; 6.56]	0.001
Other Hispanic				1.75	[1.17; 2.60]	0.008	1.73	[1.16; 2.58]	0.009
Non-Hispanic White				-	-	-	-	-	-
Non-Hispanic Black				0.95	[0.61; 1.49]	0.830	0.98	[0.62; 1.54]	0.930
Other Race^a^				1.91	[1.24; 2.93]	0.005	1.65	[1.04; 2.63]	0.035
**Education level**									
Less than 9th grade				3.62	[2.22; 5.91]	< 0.001	3.30	[2.01; 5.43]	< 0.001
9-11th grade				1.09	[0.72; 1.62]	0.706	1.07	[0.70; 1.63]	0.736
High school graduate/GED^b^									
Some college or AA degree				1.25	[0.91; 1.70]	0.160	1.37	[0.97; 1.94]	0.074
College graduate or above				2.45	[1.57; 3.84]	< 0.001	2.57	[1.63; 4.06]	< 0.001
**Energy intake (kcal)**							0.9993	[0.9991; 0.9996]	< 0.001
**Body Mass Index**									
In kg/m^2^							0.949	[0.918; 0.981]	0.003
**Diabetes Status**									
Yes							1.11	[0.72; 1.71]	0.625
No							-	-	-

All models are based on *n* = 5,416 participants. Significant regression equations were found for all 3 models: F (2,24) = 20.49 (model 1), F(12,14) = 16.96 (model 2), F (15,11) = 17.07 (model 3), respectively, with a *p*-value

^a^Includes Multi-Racial

^b^Or equivalent. *p*- < 0.001 each

In a similar style, Table 4 shows the results of the multivariate logistic regression models for potassium intake. Frequent label reading significantly increased the odds for meeting the DGA potassium recommendations (OR: 1.22, *p* = 0.015) in the crude model, whereas no significant association was observed for “rare readers”. A comparable pattern was found for model 3.

**Table 4:** Multivariate logistic regression models examining potential associations between nutrition panel reading frequency and potassium intake

**Independent variables**	OR	**CI**	**p**	OR	**CI**	**p**	OR	**CI**	**p**
	Model I			Model 2			Model 3		
**Panel reading frequency**									
Frequent readers	1.22	[1.04; 1.43]	0.015	1.14	[0.97; 1.33]	0.118	1.57	[1.29; 1.90]	< 0.001
Sometime readers	-	-	-	-	-	-	-	-	
Rare/never readers	0.88	[0.68; 1.14]	0.311	0.97	[0.76; 1.24]	0.823	0.72	[0.52; 1.01]	0.056
**Sex**									
Female				0.86	[0.66; 1.12]	0.263	3.36	[2.52; 4.48]	< 0.001
Male				-	-	-	-	-	-
**Age**									
In years				1.02	[1.01; 1.02]	< 0.001	1.03	[1.02; 1.03]	< 0.001
**Ethnicity**									
Mexican American				1.05	[0.82; 1.35]	0.688	1.01	[0.68; 1.51]	0.958
Other Hispanic				0.97	[0.75; 1.27]	0.858	1.05	[0.71; 1.57]	0.786
Non-Hispanic White				-	-	-	-	-	-
Non-Hispanic Black				0.61	[0.50; 0.74]	< 0.001	0.58	[0.44; 0.77]	0.001
Other Race^a^				1.14	[0.86; 1.51]	0.359	1.40	[0.96; 2.04]	0.075
**Education level**									
Less than 9th grade				1.39	[0.89; 2.17]	0.139	2.12	[1.39; 3.23]	< 0.001
9-11th grade				1.20	[0.92; 1.57]	0.160	1.13	[0.78; 1.63]	0.200
High school graduate/GED^b^									
Some college or AA degree				1.44	[1.12; 1.85]	0.006	1.26	[0.96; 1.67]	0.210
College graduate or above				1.70	[1.31; 2.21]	< 0.001	1.72	[1.23; 2.42]	< 0.001
**Energy intake (kcal)**							1.002	[1.002; 1.002]	< 0.001
**Body Mass Index**									
In kg/m^2^							0.99	[0.97; 1.00]	0.210
**Diabetes Status**									
Yes							0.83	[0.63; 1.11]	0.200
No							-	-	-

All models are based on *n* = 5,416 participants

^a^Includes Multi-Racial

^b^Or equivalent. Significant regression equations were found for all 3 models: F(2,24) = 8.61 (model 1), F(12,14) = 9.61 (model 2), F(15,11) = 40.22 (model 3), respectively, with a *p*-value < 0.001 for models 2 and 3 and a *p*-value of 0.002 for model 1

Table 5 displays the results of the multivariate logistic regression models for calcium intake. After adjusting for all covariates, no significant associations were observed.

**Table 5:** Multivariate logistic regression models examining potential associations between nutrition panel reading frequency and calcium intake

**Independent variables**	OR	**CI**	**p**	OR	**CI**	**p**	OR	**CI**	**p**
	Model I			Model 2			Model 3		
**Panel reading frequency**									
Frequent readers	0.99	[0.79; 1.25]	0.950	1.07	[0.81; 1.43]	0.626	1.28	[0.92; 1.76]	0.132
Sometime readers	-	-	-	-	-		-	-	
Rare/never readers	1.32	[1.01; 1.72]	0.045	1.09	[0.80; 1.48]	0.591	0.87	[0.59; 1.29]	0.481
**Sex**									
Female				0.11	[0.08; 0.13]	< 0.001	0.19	[0.14; 0.25]	< 0.001
Male				-	-	-	-	-	-
**Age**									
In years				1.01	[1.01; 1.01]	< 0.001	1.02	[1.01; 1.03]	< 0.001
**Ethnicity**									
Mexican American				0.95	[0.72; 1.27]	0.675	0.89	[0.64; 1.24]	0.483
Other Hispanic				1.00	[0.77; 1.32]	0.981	1.10	[0.78; 1.55]	0.573
Non-Hispanic White				-	-	-	-	-	-
Non-Hispanic Black				0.59	[0.48; 0.74]	< 0.001	0.59	[0.47; 0.75]	< 0.001
Other Race^a^				0.77	[0.57; 1.05]	0.074	0.79	[0.56; 1.12]	0.171
**Education level**									
Less than 9th grade				0.64	[0.41; 1.03]	0.052	0.64	[0.36; 1.12]	0.110
9-11th grade				0.84	[0.57; 1.28]	0.346	0.68	[0.43; 1.05]	0.082
High school graduate/GED^b^									
Some college or AA degree				1.16	[0.88; 1.54]	0.286	0.94	[0.70; 1.26]	0.662
College graduate or above				1.18	[0.92; 1.50]	0.187	1.06	[0.78; 1.43]	0.668
**Energy intake (kcal)**							1.001	[1.001; 1.002]	< 0.001
**Body Mass Index**									
In kg/m^2^							0.98	[0.97; 1.00]	0.056
**Diabetes Status**									
Yes							1.17	[0.83; 1.66]	0.349
No							-	-	-

All models are based on 5,416 participants

^a^Includes Multi-Racial

^b^Or equivalent. Significant regression equations were found for all 3 models: F(2,24) = 4.73 (model 1), F(12,14) = 36.17 (model 2), F(15,11) = 24.69 (model 3), respectively, with a *p*-value of < 0.001 for models 2 and 3, and a *p*-value of 0.019 for model 1

**Fig. 3:**
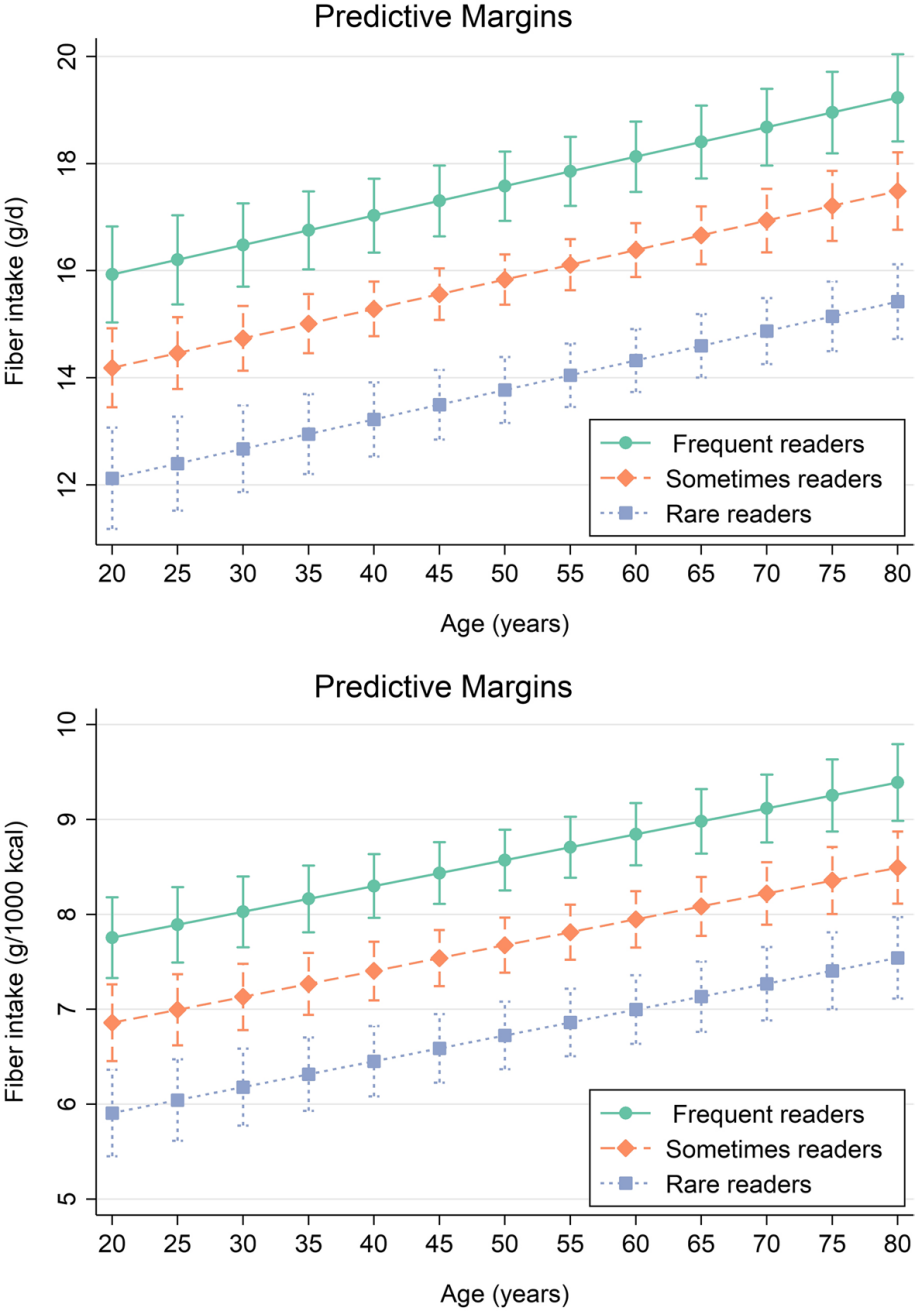
Marginsplot – mean adjusted fiber intakes by panel reading group, illustrating differences in the relationship of fiber intake and age, depending on nutrition facts panel usage

Finally, we ran linear regression models and estimated mean adjusted fiber intakes by label reading group. For this, we considered all covariates that were also used for model 3 in Table 3. Results from these models are graphically displayed in Fig. 3 and showed significant intergroup differences for both crude and energy-adjusted fiber intakes.

## Discussion

The present study sought to answer the question who actually is reading nutrition facts labels in the U.S. population, and whether reading of nutrition facts labels associated with meeting DGA recommendations of underconsumed nutrients of public health concern. The results suggest that nutrition facts panel reading associates with fiber intake, which may be relevant for public health nutrition strategies.

Fiber is one of several nutrients of public health concern and strategies to tackle “America’s fiber gap” are urgently warranted [6, 8]. The same applies to the intakes of potassium and calcium in the US, which are currently too low, with long term implications for human health [8].

Nutrition information on packaged foods may affect consumer’s dietary behavior in a way that readers are empowered to make healthier dietary choices and increase the intakes of the aforementioned nutrients [36, 39, 40]. Korean studies have demonstrated that nutrition facts panel reading is associated with an improved intake of many nutrients – particularly in men [17, 36]. Whether this applies also to U.S. adults is subject to an ongoing debate [18, 19].

Some existing studies that date back to the early-1990s suggested that the use of nutrition labels was associated with higher vitamin C intakes [19]. Ollberding et al. used data from the 2005/2006 NHANES cycle and reported that nutrition facts label usage was associated with a reduction in total energy, saturated fat and total sugar intake [23]. A reservation must be made though, that this study investigated the “old” nutrition facts label, before the redesigning by the FDA. Post et al. also used the 2005–2006 NHANES dataset and reported that those who read nutrition facts labels were significantly more likely to consume more fiber and less sugar [41].

A recent US-based study highlighted that readers of nutrition facts panels were significantly more likely to meet the national fiber intake recommendations when compared to those not reading labels (15.6% vs 6.1%, *p* < 0.001) [18]. However, these results were based on a convenience sample in young students aged approximately 22 years. More recent analyses that build on nationally representative data are scarce. This is particularly importance since some authors suggested that nutrition facts panels on packaged food products have little to no effect on average measures of diet quality [20, 21].

We addressed this ongoing controversy and investigated the associations between usage of nutrition facts panels and meeting intake recommendations for nutrients of public health concern in a large sample of over 5400 NHANES participants. Frequently reading nutrition facts labels significantly increased the odds for meeting fiber intake recommendations in all models (OR: up to 2.15 in model I). Participants rarely or never reading labels had substantially lower odds for meeting dietary fiber intake recommendations.

Our findings are thus in line with the results reported by Christoph et al., who used data from the cross sectional “Eating and Activity in Teens and Young Adults” study [16]. The authors suggested that the usage of nutrition facts panels was associated with a higher intake of fruits, vegetables and whole grains, which in turn translates into a higher fiber and potassium intake. The authors also reported a comparable set of sociodemographic variables associated with nutrition facts panel reading, and emphasized that usage was significantly higher for women, participants with higher education and those who had a better income.

Despite using another dataset, our findings are largely comparable with those from Christoph et al. [16], and reiterate that nutritions fact panels are a low-cost tool with the potential to encourage healthier eating habits. Significant associations with dietary intake were found in our study for two of three underconsumed nutrients of public health concern in the US, namely fiber and potassium. The fact that no association was found for calcium is more difficult to interpret, but does not necessarily confirm the findings by Variyam et al. and Canton-Jungles et al., who suggested that nutrition facts panels have little to no effect on diet quality [20, 21]. An aspect that could potentially play a role here is that calcium intake recommendations are higher for those who tend to read nutrition facts labels more often (e.g. older individuals and women) [8]. According to the DGA, women aged 51 + years should consume at least 1200 mg calcium per day, whereas women aged 20 years or older and men require 200 mg less.

Our findings have potential implications for public health nutrition strategies that may center around educational work and campaigns [42]. Our data suggest that nutrition facts labels could play a role in facilitating healthier dietary choices, and early education (e.g. in schools) might be valuable. The reservation must be made, however, that fiber intake was low in all groups. Even the frequent readers consumed “only” 17.09 g/d, a value well below the average intake recommendations in the DGA [8]. This demonstrates the continued need for healthcare professionals to highlight that fiber is a dietary component of public health concern, highlighting the urgent need for policy efforts to target increasing dietary fiber intake in the population [1]. In this context, it appears crucial to go beyond simply emphasizing fiber intake. An Australian study suggested that many adults knew that health professionals advocate for fiber consumption in general, but they did not understand why exactly this was the case [43]. This lack of background motivation might diminish an individual’s motivation to actually increase their intakes.

These findings are indirectly supported by a study by Gustafson et al., who conducted a nationally representative online survey of 42,018 U.S. primary shoppers in May–June 2021 about their beliefs about fiber [44]. The study revealed that over 50% of the study participants were aware of only one health benefit of fiber (or even none at all). The results also suggested that greater awareness of health benefits of fiber dramatically increased the likelihood that participant’s food choices incorporated this nutrient. Background information is thus critical and promoting increased awareness of important underconsumed nutrients may improve public health, although correct framing seems to be of utmost importance in this context [45]. Studies suggest that consumers focus more on nutrients to avoid rather than on underconsumed nutrients. Emphasizing fiber-related benefits should thus be a primary goal [45]. Point-of-decision prompts and reminder messages have been shown to be useful in this context [46, 47].

Our data suggest that nutrition facts labels may also be important, however, this analysis has several strengths and weaknesses that need to be considered.

As for the strengths, this analysis is based on a nationally-representative dataset with a large sample size (> 5400 participants). We report a topic that has been rarely investigated in U.S. adults using NHANES data and differentiate between 3 groups instead of just distinguishing readers and non-readers. We also adjusted for a variety of potential confounders that may have influenced nutrition facts panel usage. One major limitation is that we could not adjust for language issues, as variables that adequately reflect this topic were unavailable. This might be particularly the case with individuals that speak Spanish only, as labels are usually written in English. Then again, we adjusted for race/ethnicity in models 2 and 3 with the intention to consider this problem indirectly. Addition limitations include the cross-sectional nature of this study (which does not allow to determine causal relationships) and the self-reported nature of reading practices that may be prone to reporting bias. Selection bias should be particularly mentioned in this case, because the NHANES consumer behavior phone follow-up module was only conducted in a subsample of the entire 2017–2020 NHANES cycle. Despite these limitations, our data add new knowledge and contribute to a better understanding of the potential value of nutrition facts labels in U.S. adults.

## Conclusion

Nutrition facts panel reading associates with fiber and potassium but not with calcium intakes. Those participants who read labels frequently were significantly more likely to meet the DGA recommendations for fiber and potassium. Our findings thus have potential implications for public health nutrition strategies that may center around educational work. Further research, potentially in the form of controlled-prospective studies, is warranted. In the meantime, efforts to promote label reading proficiency might be a worthwhile investment.

### Supplementary Information

**Additional file 1.** STROBE Statement—Checklist of items that should be included in reports of *cross-sectional studies.*

## Data Availability

Data is publicly available online (https://wwwn.cdc.gov/nchs/nhanes/Default.aspx). The datasets used and analyzed during the current study are available from the corresponding author on reasonable request.

## Abbreviations

BMI: Body Mass Index
DGA: Dietary Guidelines for Americans
FDA: Food and Drug Administration
NCHS: National Center for Health Statistics
NHANES: National Health and Nutrition Examination Surveys
