# Supplementary material for: Nutrition facts labels: who is actually reading them and does it help in meeting intake recommendations for nutrients of public health concern?
Source: BMC Public Health. 2023 Oct 7;23:1947. doi: 10.1186/s12889-023-16859-2 (PMC10560412; doi:10.1186/s12889-023-16859-2)
Supplement: Supplementary file 1 — Additional file 1. STROBE Statement—Checklist of items that should be included in reports of cross-sectional studies. [file 12889_2023_16859_MOESM1_ESM.doc]

STROBE Statement—Checklist of items that should be included in reports of ***cross-sectional studies***

|  | Item No | Recommendation |
| --- | --- | --- |
| **Title and abstract** | 1 | (*a*) Indicate the study’s design with a commonly used term in the title or the abstract  Done on Page 2, line 30 with the term cross-sectional |
|  |  | (*b*) Provide in the abstract an informative and balanced summary of what was done and what was found  Done on Page 2, lines 35-43 where the association between nutrition facts label reading category and fiber intake are reported in two ways: with the dependent variable as a continuous outcome and as a binary outcome (meeting DGA recommendations: y/n). |
| Introduction | | |
| Background/rationale | 2 | Explain the scientific background and rationale for the investigation being reported  This is done properly on page 4, line 80. The existing studies in the field are briefly summarized. Subsequently, we conclude that the evidence for the US is mixed, and thus conducted this study using a unique dataset. |
| Objectives | 3 | State specific objectives, including any prespecified hypotheses  Done on Page 5, line 87. The two main hypotheses are presented in this paragraph. |
| Methods | | |
| Study design | 4 | Present key elements of study design early in the paper  The NHANES and its structure and summarized on page 5, lines 93 – 102 under the heading “study population and design”. |
| Setting | 5 | Describe the setting, locations, and relevant dates, including periods of recruitment, exposure, follow-up, and data collection  Same as “study design”. |
| Participants | 6 | (*a*) Give the eligibility criteria, and the sources and methods of selection of participants  Done on Page 6, lines 145 – 149 where the inclusion and exclusion criteria is listed. |
| Variables | 7 | Clearly define all outcomes, exposures, predictors, potential confounders, and effect modifiers. Give diagnostic criteria, if applicable  This is done on page 7, line 163. The outcome is discussed under the heading “underconsumed nutrients of public health concern” on page 7, followed by the covariates which are also discussed on page 6, line 136-142. Diagnostic criteria are not applicable for this type of submission. |
| Data sources/ measurement | 8* | For each variable of interest, give sources of data and details of methods of assessment (measurement). Describe comparability of assessment methods if there is more than one group  See above, all NHANES modules are explained and listed in the methods section on pages 5 and 6. |
| Bias | 9 | Describe any efforts to address potential sources of bias  Bias are inherent to cross-sectional studies and cannot be removed. We acknowledge this in the limitation section on page 11, line 303 pp. |
| Study size | 10 | Explain how the study size was arrived at  Done in Figure 1, participant inclusion flowchart. |
| Quantitative variables | 11 | Explain how quantitative variables were handled in the analyses. If applicable, describe which groupings were chosen and why  This has already been done in the variable section. All quantitative outcome variables were considered as continuous, with the exception for the logistic regression models were the DGA cut-offs were employed. This is explained in detail on page 5, line 122-126. |
| Statistical methods | 12 | (*a*) Describe all statistical methods, including those used to control for confounding  Done on page 7, lines 150-174. |
|  |  | (*b*) Describe any methods used to examine subgroups and interactions  Not applicable |
|  |  | (*c*) Explain how missing data were addressed  Participants with missing data were excluded. This has already been described in detail in the inclusion- and exclusion criteria section. |
|  |  | (*d*) If applicable, describe analytical methods taking account of sampling strategy  Not applicable |
|  |  | (*e*) Describe any sensitivity analyses  Not applicable |
| Results | | |
| Participants | 13* | (a) Report numbers of individuals at each stage of study—eg numbers potentially eligible, examined for eligibility, confirmed eligible, included in the study, completing follow-up, and analysed  Figure 1. |
|  |  | (b) Give reasons for non-participation at each stage  Figure 1. |
|  |  | (c) Consider use of a flow diagram  Figure 1. |
| Descriptive data | 14* | (a) Give characteristics of study participants (eg demographic, clinical, social) and information on exposures and potential confounders  Table 1 and page 8, lines 184-190. |
|  |  | (b) Indicate number of participants with missing data for each variable of interest  Not applicable as those participants were excluded. |
| Outcome data | 15* | Report numbers of outcome events or summary measures  Done on page 8, line 194-201. Also found in Tables 2-5. |
| Main results | 16 | (*a*) Give unadjusted estimates and, if applicable, confounder-adjusted estimates and their precision (eg, 95% confidence interval). Make clear which confounders were adjusted for and why they were included  Done on page 8, line 205- page 9, line 230. Also found in Tables 3 – 5, where the models can be clearly identified. |
|  |  | (*b*) Report category boundaries when continuous variables were categorized  This was not the case here. |
|  |  | (*c*) If relevant, consider translating estimates of relative risk into absolute risk for a meaningful time period  Not applicable |
| Other analyses | 17 | Report other analyses done—eg analyses of subgroups and interactions, and sensitivity analyses  Not applicable |
| Discussion | | |
| Key results | 18 | Summarise key results with reference to study objectives  Done on page 10, lines 256 – 261. |
| Limitations | 19 | Discuss limitations of the study, taking into account sources of potential bias or imprecision. Discuss both direction and magnitude of any potential bias  Done on page 11, lines 305 – 314. |
| Interpretation | 20 | Give a cautious overall interpretation of results considering objectives, limitations, multiplicity of analyses, results from similar studies, and other relevant evidence  Done on page 10, lines 262 – 268 |
| Generalisability | 21 | Discuss the generalisability (external validity) of the study results  This item is not applicable for our submission. Since we use nationally representative U.S. data, it is intrinsically clear what our data stands for. |
| Other information | | |
| Funding | 22 | Give the source of funding and the role of the funders for the present study and, if applicable, for the original study on which the present article is based  We received no funding. Open Access publishing enabled by Project DEAL as discussed on page 13, lines 351-352. |

*Give information separately for exposed and unexposed groups.

**Note:** An Explanation and Elaboration article discusses each checklist item and gives methodological background and published examples of transparent reporting. The STROBE checklist is best used in conjunction with this article (freely available on the Web sites of PLoS Medicine at http://www.plosmedicine.org/, Annals of Internal Medicine at http://www.annals.org/, and Epidemiology at http://www.epidem.com/). Information on the STROBE Initiative is available at www.strobe-statement.org.
